# SUPER-FOCUS: a tool for agile functional analysis of shotgun metagenomic data

**DOI:** 10.1093/bioinformatics/btv584

**Published:** 2015-10-09

**Authors:** Genivaldo Gueiros Z. Silva, Kevin T. Green, Bas E. Dutilh, Robert A. Edwards

**Affiliations:** ^1^Computational Science Research Center; ^2^Department of Biology, San Diego State University, San Diego, CA 92182, USA; ^3^Theoretical Biology and Bioinformatics, Utrecht University, 3584 CH, Utrecht, The Netherlands; ^4^Centre for Molecular and Biomolecular Informatics, Radboud Institute for Molecular Life Sciences, Radboud University Medical Centre, 6525 GA, Nijmegen, The Netherlands; ^5^Department of Marine Biology, Institute of Biology, Federal University of Rio de Janeiro, Brazil; ^6^Department of Computer Science, San Diego State University, San Diego, CA 92182, USA; ^7^Division of Mathematics and Computer Science, Argonne National Laboratory, Argonne, IL 60439, USA

## Abstract

**Summary:** Analyzing the functional profile of a microbial community from unannotated shotgun sequencing reads is one of the important goals in metagenomics. Functional profiling has valuable applications in biological research because it identifies the abundances of the functional genes of the organisms present in the original sample, answering the question what they can do. Currently, available tools do not scale well with increasing data volumes, which is important because both the number and lengths of the reads produced by sequencing platforms keep increasing. Here, we introduce SUPER-FOCUS, SUbsystems Profile by databasE Reduction using FOCUS, an agile homology-based approach using a reduced reference database to report the subsystems present in metagenomic datasets and profile their abundances. SUPER-FOCUS was tested with over 70 real metagenomes, the results showing that it accurately predicts the subsystems present in the profiled microbial communities, and is up to 1000 times faster than other tools.

**Availability and implementation:** SUPER-FOCUS was implemented in Python, and its source code and the tool website are freely available at https://edwards.sdsu.edu/SUPERFOCUS.

**Contact:** redwards@mail.sdsu.edu

**Supplementary information:** Supplementary data are available at *Bioinformatics* online.

## 1 Introduction

Prokaryotes and the viruses that infect them are the most abundant organisms on earth (Whitman *et al.*, 1998), and it is important to understand both who they are and what they are doing. In many environments, the majority of the microbes cannot be cultured by using standard laboratory techniques, and metagenomics is the preferred way to study them as a whole community (Handelsman, 2004). Next-generation DNA sequencing (NGS) technologies have sped up the sequencing process, reduced the cost, increased the sampling resolution and opened new horizons in the biological sciences (Zhang *et al.*, 2011).

Understanding microbial communities is important in many areas of biology. For example, metagenomes can distinguish taxonomic and functional signatures of microbes associated with humans (Consortium, 2012), sponges (Trindade-Silva *et al.*, 2012, 2013), red seaweed (Oliveira *et al.*, 2012) and diseased and healthy states of corals (Garcia *et al.*, 2013).

Functional annotation of metagenomic reads normally requires the alignment of sequences to a large database of annotated sequences to identify homologs (Mendoza *et al.*, 2015). There are many databases for annotations at the functional system or pathway level, including the SEED (Overbeek *et al.*, 2005) which contains subsystems (sets of protein families with a similar function), and the large metabolic pathway databases KEGG (Kanehisa and Goto, 2000) and MetaCyc (Caspi *et al.*, 2010).

Many of the tools for functional profiling are slow (Lindgreen *et al.*, 2015), suggesting that there is an opportunity for improvements of these tools. Currently, available tools generally use either homology, i.e. by aligning the metagenomic sequencing reads against an annotated reference database, or use exact matches (k-mers) to link metagenomic sequencing reads to the annotated sequences. Homology-based metagenome annotation tools programs are still frequently based on pre-NGS algorithms such as BLAST (Altschul *et al.*, 1997) or BLAT (Kent, 2002) to identify the best hit in a large database, although new homology search algorithms such RAPSearch2 (Zhao *et al.*, 2012) and DIAMOND (Buchfink *et al.*, 2015) have recently been developed to reduce the run time. MG-RAST (Meyer *et al.*, 2008) and MEGAN 5 (Mitra *et al.*, 2011) both align sequences to a reference database to profile the metagenomic sample. MG-RAST first predicts the open reading frames (ORFs) on the metagenomic sequence using FragGeneScan (Rho *et al.*, 2010) and then aligns the translated amino acid sequences to the M5NR database via BLAT. MEGAN accepts as input the tabular results files created from programs such as blastx/blastp, DIAMOND, or RAPSearch2 to the NR database, and creates taxonomic and/or functional profiles based on the search output.

Exact match or k-mer based approaches use oligonucleotides to identify the hits in a metagenome. For example, real time metagenomics (RTMg) (Edwards *et al.*, 2012) identifies all words of length *k* (where *k* is typically between 7 and 12 amino acids) that are a unique signature for a set of functionally related proteins, and uses them to profile the functions present in the metagenomic sample. This approach has also been successfully applied to assign taxonomic labels to metagenomic sequences using a length of *k* of 31 nt (Wood and Salzberg, 2014; Ounit *et al.*, 2015).

We developed a novel approach, named SUPER-FOCUS, which classifies each sequence in the metagenome into a subsystem. SUPER-FOCUS aligns all the input data against a reduced database with contains only the subsystems present in the organisms in that metagenome. The speed up derives from three improvements compared with the standard metagenome annotation pipelines. First, the SEED database was clustered using CD-HIT (Huang *et al.*, 2010) using a similar approach as previously discussed and applied to the Genbank NR database (Li *et al.*, 2012); second, the metagenomic query sequences are profiled using FOCUS (Silva *et al.*, 2014), an ultra-fast tool that identifies the organisms in the metagenome; and finally, comparisons are performed using RAPSearch2 which is ∼2–3 times faster than BLAT and 100 times faster than blastx, but has no reduction in sensitivity or specificity when compared with BLAST (Berendzen *et al.*, 2012). We compare the performance of SUPER-FOCUS to RTMg, MEGAN and MG-RAST using different reduced databases and over 70 metagenomic datasets of different sizes and from different environments. The results shows that SUPER-FOCUS speeds up the process of sequence functional annotation 37, 60 and 1000 times faster than RTMg, MEGAN and MG-RAST, respectively. We apply SUPER-FOCUS to a novel dataset from a coral reef environment and show that the taxonomy is conserved across islands while functions adapt to local conditions.

## 2 Methods

**Fig. 1.**
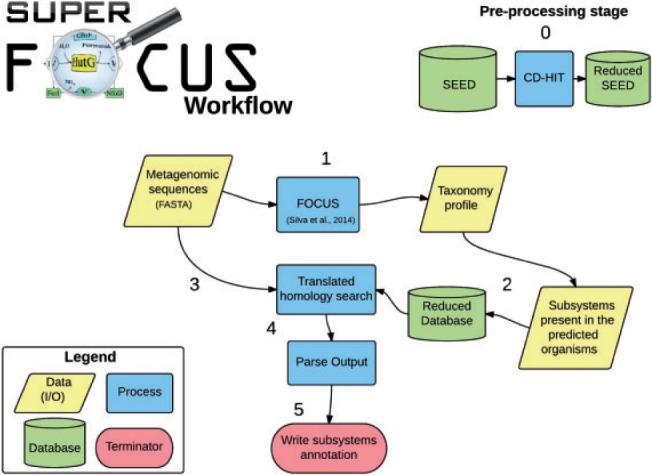
Workflow of the SUPER-FOCUS program

The SUPER-FOCUS workflow consists of one pre-processing step (step 0) and five processing computational stages which are represented in Fig. 1 and described below:

1. Create reduced databases of subsystems using CD-HIT with different parameters.
2. Identify the genera present in the metagenomic sample using FOCUS.
3. Select subsystems present in the predicted organisms to create a new reduced database.
4. Align input data against the reduced database using RAPSearch2 (default aligner), DIAMOND, or blastx.
5. Parse the alignment output and keep best-hit(s) with, e.g. maximum E-value 1e^−5^, minimum 60% identity, and minimum alignment length 15 amino acids (these are all default values that can be changed). If more than one best hit is found per query sequence, the program keeps all the subsystems for all the best hits.
6. Write functional annotations for subsystems levels 1–3.

### 2.1 Reduced reference dataset

The SEED database was developed by members of the Fellowship for Interpretation of Genomes (FIG) and Argonne National Laboratory starting in 2004 (Overbeek *et al.*, 2004) with the goal of clustering sets of proteins which implement a specific biological process or structural complex into subsystems.

**Fig. 2.**
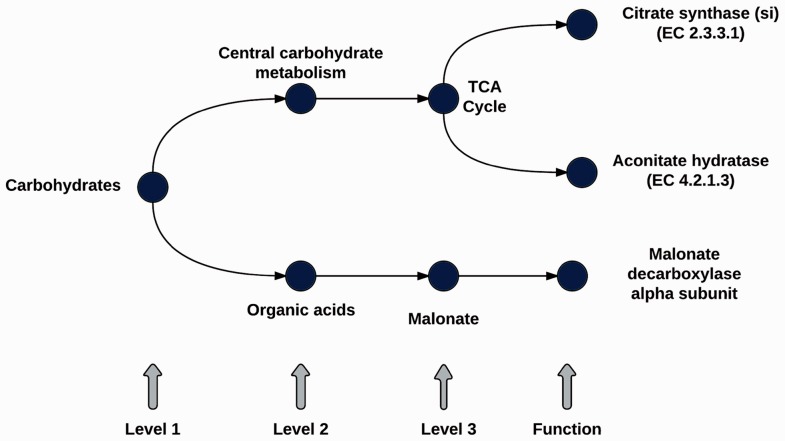
Representation of a subsystem structure (Levels 1–3 classifications and Function)

The database is composed of subsystems structured into three levels, the first level is the most general class and the third level is the more specific class, and one functional role (Fig. 2 for an example of one subsystem).

SUPER-FOCUS requires a group of reference sequences to identify the subsystems present in a metagenome. The proteins annotated into 1290 subsystems (Supplementary Table S1) were downloaded from the SEED servers (Aziz *et al.*, 2012) using the SEED API (Disz *et al.*, 2010) on December 12, 2014 and all identical proteins were removed. Next, the proteins within each subsystem were clustered separately using CD-HIT in order to cluster exact and nearly identical protein sequences (the clustering was repeated four times with sequence identity cut-off: 100, 98, 95 and 90%) reducing the total reference dataset, respectively, from 3.1 to 3 GB (100% identity clustering; ∼6% sequence reduction), 2.1 GB (98% identity clustering; ∼30% sequence reduction), 1.8 GB (95% identity clustering; ∼40% sequence reduction) and 1.6 GB (90% identity clustering; ∼52% sequence reduction).

SUPER-FOCUS databases were named by the identity used in their clustering e.g: DB_100 used 100% sequence identity, DB_98 (default database) used 98% identity, DB_95 used 95% identity, and DB_90 used to 90% identity in clustering. Due to the reduction of the reference database into non-redundant clusters, a slight loss of functional resolution is lost. We recommend that the database choice should be made depending on how specific the functional profiling is needed. For example, if the function level is needed, DB_100 is the recommended. Otherwise, the other databases can be used with a little loss of sensitivity and high precision as shown later.

### 2.2 Aligner choice

RAPSearch2 is the default aligner for SUPER-FOCUS because it has a reduced memory requirement of ∼2 GB RAM when running in single threaded mode and around 3.5 GB when running in four-thread mode.

DIAMOND (Buchfink *et al.*, 2015) has a large memory requirement of ∼100 GB RAM for these datasets. Any aligner that provides a tabular output can be easily integrated to the SUPER-FOCUS pipeline; e.g. BLAST, or DIAMOND which is ∼20 times faster than RAPSearch2 for large datasets but slower than blastx for small datasets. RAPSearch2 and DIAMOND have a fast version, which is less sensitive, but 900 and 20 000 times faster than blastx, respectively. SUPER-FOCUS was tested with both the fast and sensitive settings for these algorithms.

### 2.3 The presence or absence of subsystems in complete genomes

We downloaded all the encoded proteins in complete bacterial and archael genomes, including any integrated phophages or plasmids, from the Genbank database on March 12, 2015 (ftp://ftp.ncbi.nih.gov/genomes/Bacteria). To identify the presence of SEED subsystems within these genomes, we aligned the proteins to the complete SEED database using blastp 2.2.25+ targeting the 250 top hits. Only the best hit(s), i.e. those with the lowest E-value, were kept and used to stipulate which subsystems were present in each genome (maximum E-value: 1e-5, minimum identity: 60%, minimum alignment length: 15 amino acids, and Supplementary Fig. S1 shows that the used hits had 361.67 ± 248.14 of alignment length, which suggests that ORF’s were correctly assigned into a subsystem.

The presence of subsystems in each genus was determined via the assignments of each subsystem at the species level (Supplementary Table S2), and this presence/absence matrix is used to further reduce the database in step 2 of SUPER-FOCUS pipeline.

We also annotated the same genomes using RAST (Aziz *et al.*, 2008), and on Supplementary Material we show the comparison between the presence/absence matrices from RAST and blastp generate a similar results.

### 2.4 Testing set

In order to evaluate SUPER-FOCUS performance, both public and private real metagenomic datasets were selected as test cases:

**Public data:** Fifty datasets (2.2 GB) from six different human body sites sequenced by the Human Microbiome Project (HMP) (Consortium, 2012) were selected as a test case (Supplementary Table S3) and three marine viral metagenomes (total of ∼500 MB) from the Moore Marine Phage/Virus Metagenomes project (CAM_PROJ_BroadPhage) (Supplementary Table S4) were analyzed using SUPER-FOCUS. Human contamination was removed from all the testing sets using DeconSeq (Schmieder and Edwards, 2011).

**Private data:**

**Study sites**

One big data metagenome was generated from a simulated colon bioreactor, totaling 7.7 GB. Twenty coral metagenomics samples were collected from February 2010 to September 2013 across the Central and Southern Pacific Ocean as part of the National Oceanic and Atmospheric Administration (NOAA) Coral Reef Ecosystem Division (CRED) monitoring program (Supplementary Table S5).

**Sample collection**

Seawater was collected on coral reefs across the Pacific Oceans as described in Haas *et al.* (2014). Briefly, 2.2 l of water was collected in diver-deployed Niskin bottles at ∼10 m depth within 30 cm of the benthos at each site. Sample water was then flushed through a 0.22 μm Sterivex filter. After filtration, excess water was flushed out of the Sterivex using a clean 10 ml syringe filled with air. Sterivexes were then labeled and placed back into the original package, sealed with tape and stored at −20°C until extraction.

**DNA extraction and sequencing**

Total DNA was extracted from a 0.22 μm Sterivex filter using the Nucleospin Tissue Kit (Macherey-Nagel, Dueren, Germany) following manufacturer’s protocol. Briefly, filters were thawed and excess water was removed by flushing the water out with 10 ml Lure-Lok syringe. One end of the filters were sealed with Parafilm and 410 μl of T1 lysis buffer with 20 mg/ml Proteinase K was added into each filter from the other end. The end of the filters were sealed and placed into a 55°C oven on a rotating spit overnight. After incubation, 400 μl of Buffer B3 was added to each filter and placed back in the rotating oven at 70°C for 30 min. The lysate was retrieved from the filter using a 3 ml Lure-Lok syringe and placed into a new 1.5 ml microcentrifuge tube. Four hundred twenty micrometer liters of 100% ethanol was added into each tube containing the lysate and DNA was recovered described in the manufacture’s protocol. DNA concentration was measured using the Qubit High Sensitivity dsDNA kit (Life Technologies, NY) and DNA purity was evaluated using NanoDrop (Thermo Scientific). The Nextera XT DNA Library Prep Kit (Illumina, CA) was used for sequence library preparation and the manufactures protocol was followed. In short, samples were diluted to 0.2 ng/µl and a total of 1 ng of DNA from each sample was processed. DNA was amplified via a limited-cycle PCR program, AMPure XP beads (Beckman Coulter, CA) were used for purification and for size selection (> 500 bp) of the DNA. 11 samples were tested using the 2100 Bioanalyzer (Agilent Technologies, CA) to ensure size selection was successful. The size-selected samples were then sequenced on the Illumina MiSeq platform (Illumina, CA) using the MiSeq Reagent Kit v3.

### 2.5 Sensitivity, precision and speed of SUPER-FOCUS analysis

To benchmark our annotation pipeline with real metagenomes, we had to define a true annotation of the metagenomic sequencing reads. Because blastx searches the DNA sequences in protein space, we consider it to be the most sensitive search tool, and we defined the true annotation of a metagenomic sequencing read as the best hit(s) of a blastx search of the read against the complete database (DB_100), and if there is more than one best hit with an equally low E-value, all are used. Thus, for a given functional level (e.g. Subsystem level 1, 2 or 3), sensitivity is defined as the ratio between the number of correct assignments by SUPER-FOCUS and the total number of sequences annotated by a blastx search against DB_100 (‘true answer’), and precision is defined as the ratio between the number of correct assignments by SUPER-FOCUS and the total number of classified sequences by SUPER-FOCUS.

The speed of SUPER-FOCUS analysis, measured in thousands of sequences analyzed per minute, was estimated by timing the run time in seconds for each metagenome using the python library time, and dividing the time by the total number of sequences in the metagenome; it estimates the number of seconds to align each sequence in the metagenome against the target database (e.g. SEED or NR).

## 3 Results and discussion

### 3.1 Validation of clustered database and SUPER-FOCUS evaluation

Prior to testing SUPER-FOCUS we independently validated our database construction and size reduction. The HMP and viromes testing set metagenomes were aligned against the SUPER-FOCUS database DB_100 using blastx, as described in the methods, and each query sequence was assigned to a subsystem using the SUPER-FOCUS best-hit method. Next, DB_100 blastx's assignments were assumed to be the right answer, and same testing sets were aligned against DB_100, DB_98, DB_95 and DB_90, but now using RAPSearch2 as the aligner with the same parameters previously used with blastx. RAPSearch2 was run with 24 threads in the sensitive and fast modes using the SUPER-FOCUS workflow. RAPSearch2 without the SUPER-FOCUS workflow using database DB_100 represents how a regular user would profile a metagenomic dataset only using the complete SEED and a fast aligner. This analysis was added to show that part of the loss of sensitivity and precision from SUPER-FOCUS profiling comes from RAPSearch2.

**Fig. 3.**
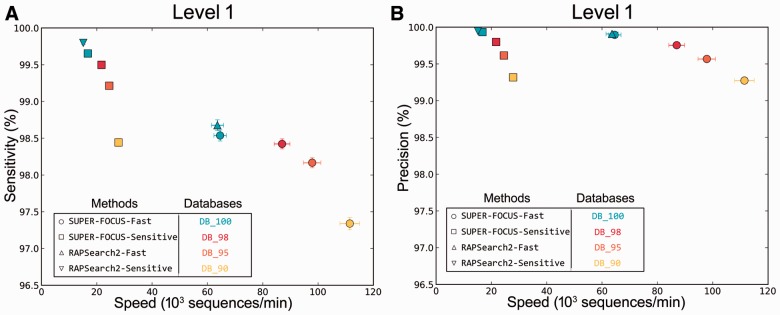
Percent classification sensitivity (**A**) and precision (**B**) of level 1 subsystems and speed of RAPSearch2 and SUPER-FOCUS using different databases and parameter modes. This analysis was based on a comparison of 50 HMP metagenomes, where blastx assignments using DB_100 were considered to be the true answer

For the 50 HMP metagenomes with short reads, we measured both the sensitivity (Fig. 3a for sensitivity using subsystem level 1 classification, Supplementary Fig. S2 for level 2 and 3 classifications) and precision (Fig. 3b) for precision using subsystem level 1 classifications, Supplementary Fig. S3 for level 2 and 3 classifications.

We used blastx as our notion of ‘truth’ as it is the most widely used algorithm in metagenomics analysis. For the 50 HMP metagenomes the average processing time (sequences/minute) is compared with the sensitivity at each of the three subsystem levels. The more sensitive the profile, the longer it is going to take, and the more broader the categories (subsystems level 1), the more sensitive the results are.

**Fig. 4.**
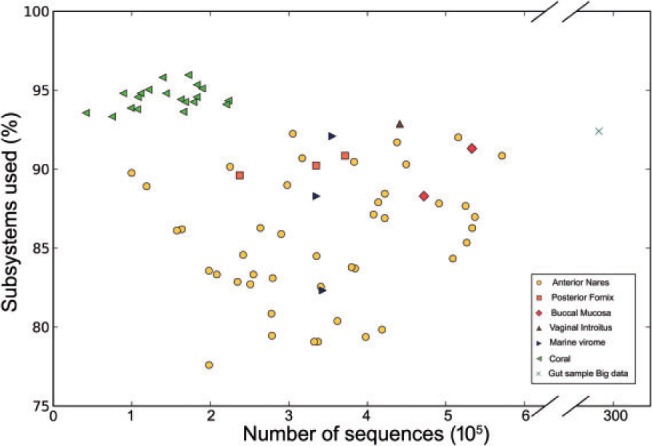
Percentage of level 3 subsystems present in all the testing set metagenomes predicted by SUPER-FOCUS

Figure 3 also shows that the SUPER-FOCUS approach was slightly faster, but less sensitive when compared with RAPSearch2 without the SUPER-FOCUS workflow alignments; it was faster because on average 88.53 ± 5% of the 1290 subsystems in the SEED database were used to profile all the metagenomes in the testing set (Fig. 4) in step 2 of the SUPER-FOCUS pipeline, and the misclassified sequences explain the loss of sensitivity as described later.

Overall, there was <1% loss of sensitivity (Supplementary Fig. S4a) and precision (Supplementary Fig. S4b) between all the levels, which shows the efficiency of using the clustered database presented in this paper and the SUPER-FOCUS approach compared with blastx searches.

### 3.2 Understanding SUPER-FOCUS misclassifications

**Fig. 5.**
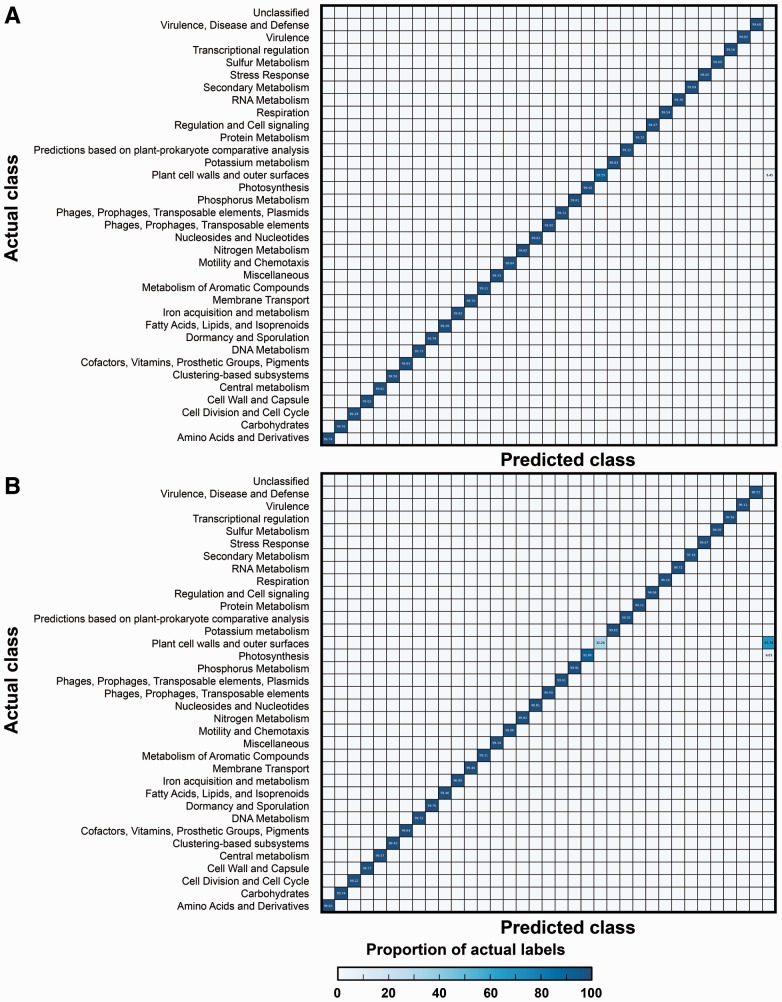
Confusion matrix displaying the percentage of correct assignments in each level 1 subsystem for the 50 HMP metagenomes. (**a**) Shows the RAPSearch2 assignments in the sensitive mode to DB_100. (**b**) Shows the SUPER-FOCUS assignments in the sensitive mode to DB_100

In order to understand the SUPER-FOCUS misclassifications when compared with RAPSearch2 without the SUPER-FOCUS workflow, two confusion matrices were generated to compare predicted and real sequence annotations: Figure 5a presents the RAPSearch2-Sensitive results using the DB_100, and the only significant loss of sensitivity was 6.45% of sequences that were supposed to be classified into the ‘Plant cell walls and outer surfaces’ subsystems but were not classified; this loss of sensitivity is explained because RAPSearch2 is based on a reduced amino acid alphabet of 10 symbols, which makes the tools less sensitive, while blastx uses a complete amino acid alphabet. Figure 5b shows the results for SUPER-FOCUS-Sensitive using DB_100, and now 67.74% of the sequences that were supposed to be classified into the ‘Plant cell walls and outer surfaces’ subsystems and 6.03% of the sequences that were supposed to be classified into the ‘Photosynthesis’ subsystems were not classified.

To investigate the SUPER-FOCUS misclassifications, those sequences were aligned against the NR-database using blastx and an E-value cutoff of 1 e^−^^5^. The best-hit was selected for each sequence, and the taxonomic classification for each hit was recovered using Biopython (Cock *et al.*, 2009).

The results show that ∼25% of the wrong assignments were from the Eukaryota and ∼1.3% of the wrong assignments were derived from viruses. The rest of the misclassified sequences were associated with Bacteria. Ninety-nine percentage of those were not identified by FOCUS and 78% were not identified by MetaPhlAn because neither FOCUS nor MetaPhlAn include those bacteria in their databases. Of the 22% that were identified by MetaPhlAn (used to analyzed the data in the HMP paper) (Segata *et al.*, 2012), just over half of those (57%) had <2% relative abundance, suggesting that both FOCUS and MetaPhlAn are missing the rare species in the environment (Supplementary Table S6).

SUPER-FOCUS uses FOCUS in its pipeline, a tool that was developed to taxonomically profile microbial data. The FOCUS database only contains bacterial and archaeal genomes, which explains the misclassification of metagenomic reads from other microbial clades. We showed that the real metagenomes used herein contained viral and eukaryotic sequences, even after the sequences were filtered. For example, while human contamination from the HMP data was already removed using BMTagger (Rotmistrovsky and Agarwala, 2011), we were still able to identify human reads with the DeconSeq tool (Schmieder and Edwards, 2011). These contaminations affect biological conclusions (Weiss *et al.*, 2014) and lead to increased computing time.

**Fig. 6.**
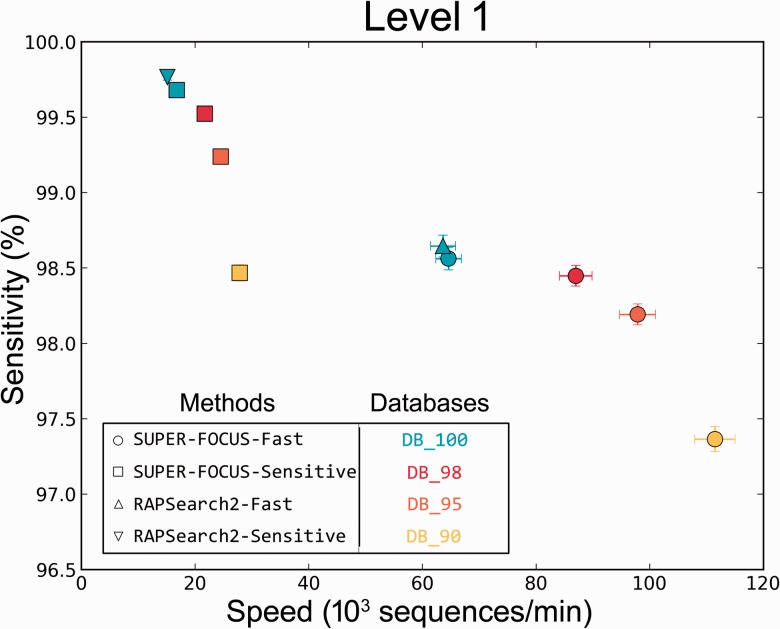
Classification sensitivity using level 1 classifications and speed comparison of 50 HMP metagenomes using RAPSearch2 and SUPER-FOCUS using different databases and modes, but removing Eurkaryota and viral assignments. blastx assignments using DB_100 were considered to be the true answer

SUPER-FOCUS was designed to only classify microbial data. The SUPER-FOCUS pipeline guarantees a more accurate microbial functional analysis and does not classify eukaryotic or viral sequences. Thus, if hits to the Eukaryotic and Viral Kingdoms are ignored, the SUPER-FOCUS approach would present a better profile than the one present in Fig. 3a as shown in Fig. 6 for sensitivity at level 1, Supplementary Fig. S5a for level 2 and 4 (b) for level 3.

### 3.3 Comparison of SUPER-FOCUS with other tools

SUPER-FOCUS was compared with RTMg, MEGAN and MG-RAST, and all the four tools were tested using default parameters. MEGAN 5.10 and SUPER-FOCUS were run using default parameters and their default reference database, either online (MG-RAST and RTMg) or using one core on a server with 24 processors × 6 cores Intel(R) Xeon(R) CPU X5650 @ 2.67 GHz and 189 GB RAM. MEGAN uses the NR database, and it was downloaded on January 22, 2015. MG-RAST uses the M5NR as database, and its last update is not known because MG-RAST does not provide that information. RTMg is a web server which uses the SEED as database and was updated on November 8, 2013.

**Fig. 7.**
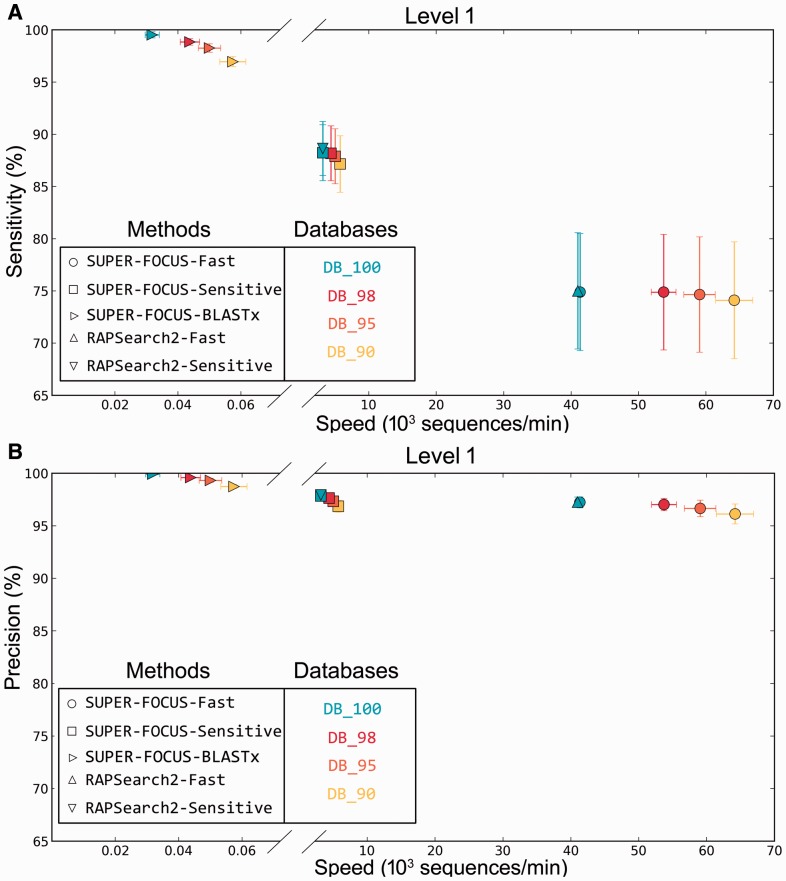
Classification sensitivity (**a**) and precision (**b**) percent using level 1 and speed comparison of three viromes using RAPSearch2 and SUPER-FOCUS using different databases and modes. blastx assignments using DB_100 were considered to be the true answer

For the three viromes that were sequenced using 454 technology and thus had longer reads, SUPER-FOCUS sensitivity and precision were evaluated against the annotations based on the blastx searches against DB_100 as the true assignments, and its runtime was compared with MEGAN, MG-RAST and RTMg. Here, RAPSearch2’s performance was tested using different numbers of threads (24, 18, 12 and 6) (Supplementary Fig. S6), blastx was also used to align the sequences because RAPSearch2 is known to be less sensitive for 454 data (Buchfink *et al.*, 2015), and because DIAMOND was designed for large datasets, and it is slower than blastx for small datasets. RAPSearch2 is less sensitive for long sequences as shown in Fig. 7a. SUPER-FOCUS had a high precision, using all the databases, of ∼98.4 ± 1% using blastx with level 1 classifications (Fig. 7b). Subsystems sensitivity and precision measurements for level 2 and 3 subsystem classifications are shown, respectively, in Supplementary Figs S7 and S8. BLAST is slow, thus the blastx results for Fig. 7 was generated in a cluster at the San Diego State University facilities, and the run time was approximated based on the knowledge that RAPSearch2 is ∼100 times faster than blastx.

**Fig. 8.**
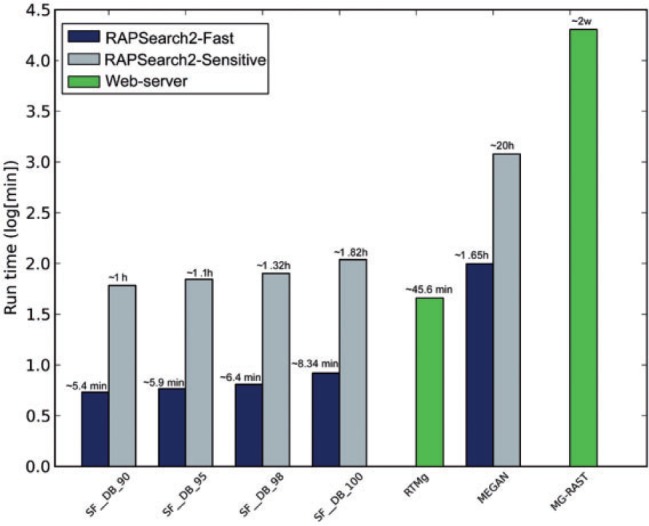
Run time comparison for the three marine viromes using SUPER-FOCUS, RTMg, MEGAN and MG-RAST

In the timing tests, SUPER-FOCUS had the fastest profiling using RAPSearch2 in its fast mode. RTMg was faster than RAPSearch2 sensitive (Fig. 8). MEGAN can be enhanced by using RAPSearch2, and the underlying framework hinders MG-RAST.

Interestingly, despite the absence of phages in the FOCUS database, SUPER-FOCUS was able to predict correctly the subsystems in the viromes. We hypothesize that FOCUS predicted the microbial host for the phages, and from those genera that carry the subsystems present in the viral metagenomes. Phages are very diverse which means that the subsystems associated with phages (‘Phages, Prophages, Transposable elements, Plasmids’, ‘Virulence’ and ‘Virulence, Disease and Defense’), did not cluster well in the SUPER-FOCUS database creation. This lack of clustering explains why the sensitivity across different databases does not change much in Fig. 7.

**Fig. 9.**
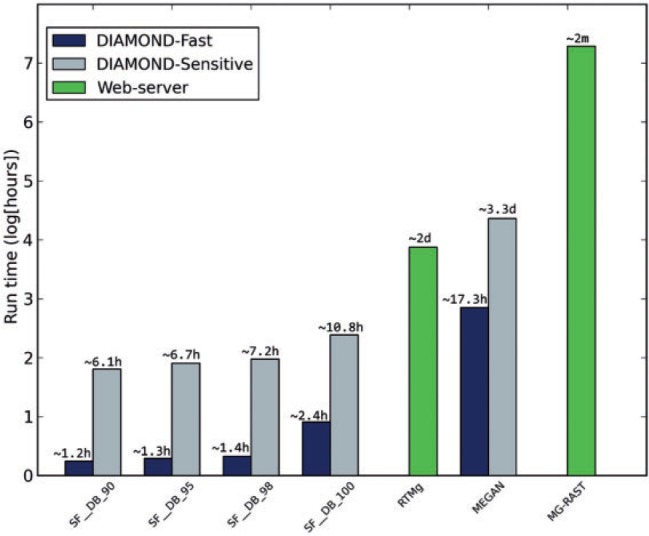
Run time comparison for the one big data metagenome using SUPER-FOCUS, RTMg, MEGAN and MG-RAST

**Fig. 10.**
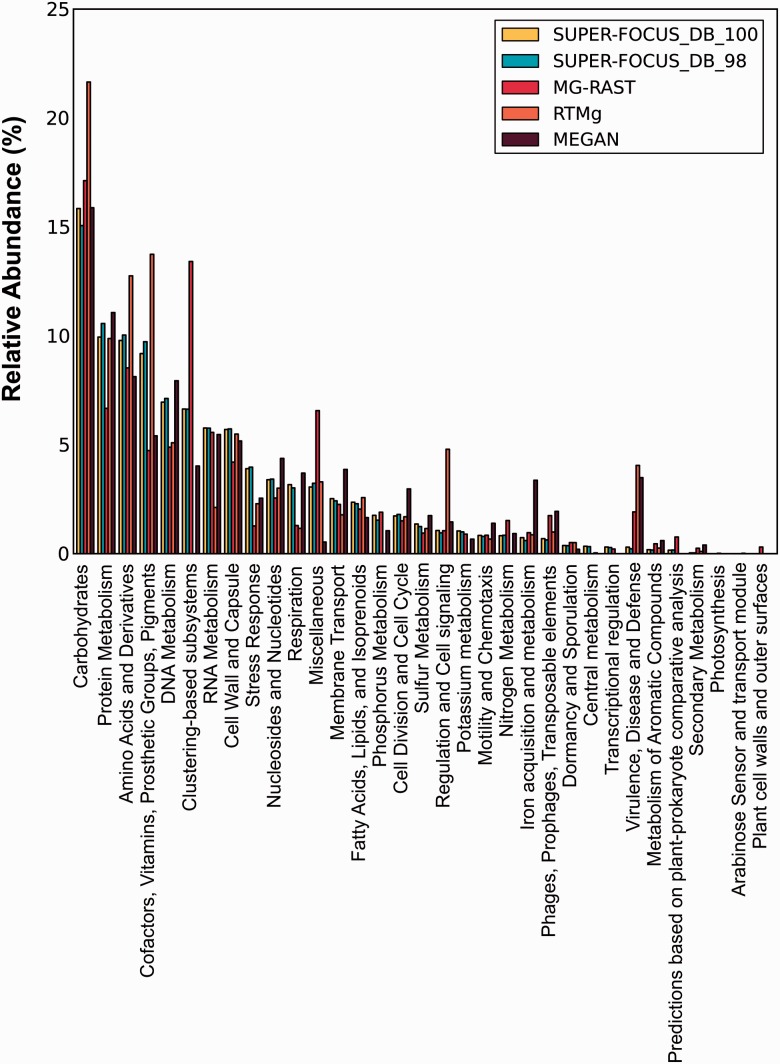
Comparison of level 1 subsystems profile of one big data metagenome using SUPER-FOCUS, RTMg, MEGAN, MG-RAST and blastx that are considered to be the true answer

For the one big data metagenome analyzed here, the runtime of SUPER-FOCUS was only compared with MEGAN, MG-RAST and RTMg, and due to the large number of sequences (30 917 457 reads), DIAMOND was used as default aligner for the SUPER-FOCUS and MEGAN tools. As shown in Fig. 9, SUPER-FOCUS was the fastest tool followed by RTMg, MEGAN, and then MG-RAST. It is important to point out that SUPER-FOCUS (and MEGAN) used 24 threads, and even if the program had been set to use less threads, it would be still expected to be faster than RTMg because as shown in Fig. 8 SUPER-FOCUS is ∼4.4 (most sensitive) and 37.7 (fastest) times faster than RTMg. The SUPER-FOCUS profiling was compared with MEGAN, RTMg and MG-RAST as shown in Fig. 10, and the results show that the three tools are comparable to each other, except to MG-RAST which apparently over predicted hits to the ‘Clustering-based subsystems’ and RTMg which did not report any hits this subsystem as they are ignored.

### 3.4 Coral metagenomes functional profiling

**Fig. 11.**
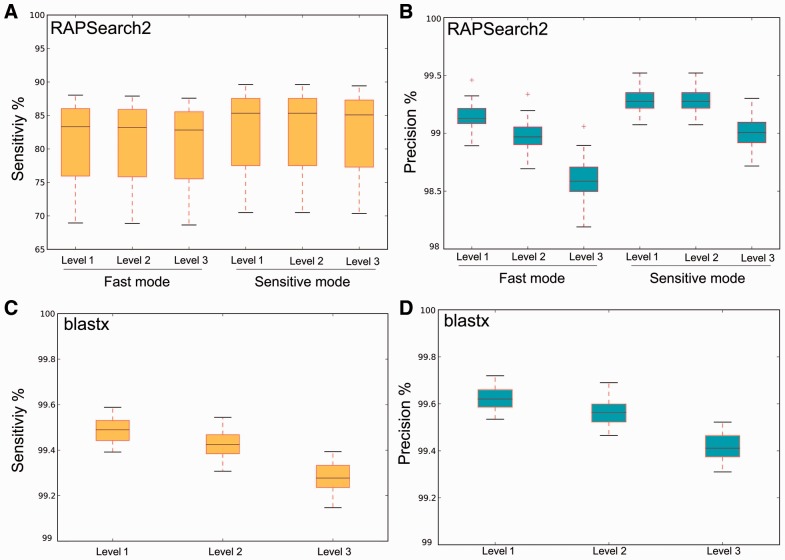
Box plots displaying the percent sensitivity (**A** and **C**) and precision (**B** and **D**) of RAPSearch2 (**A** and **B**), blastx (**C** and **D**) annotation of the 20 coral metagenomes. RAPsearch2 was tested in the fast and sensitive modes

Twenty coral metagenomes from four sites were also analysed to test the robustness of using SUPER-FOCUS in the marine environment. First, the coral sequences were profiled using FOCUS, which is part of SUPER-FOCUS, to understand the taxonomic profile among the sites; and second, the same sequences were classified into subsystems using SUPER-FOCUS with RAPSearch2 and DB_98 as database which we have already shown to be sensitive and precise. In addition to RAPSearch2, blastx was also added to the analysis because the sequenced reads average ∼218 ± 64.4 bp and RAPSearch2 is known to be less sensitive for reads longer than 100 bp (Buchfink *et al.*, 2015). The difference in sensitivity for the long reads coral data between blastx and RAPSearch2 is shown in Fig. 11a. The median RAPSearch2 sensitivity was ∼83%, although it was still precise (∼99%; Fig. 11b); in comparison, the median blastx sensitivity was (∼99.5%) and precision was (∼99.5%) as shown in Fig. 9c and d.

**Fig. 12.**
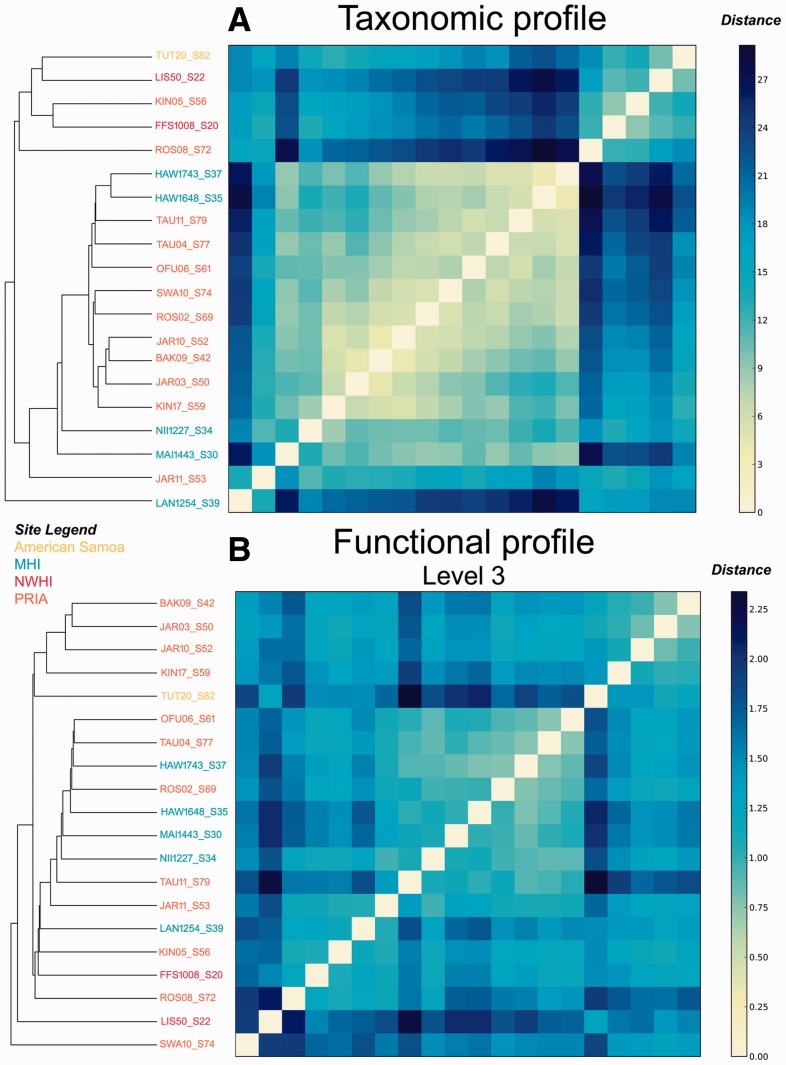
Hierarchical clustering of the taxonomic (**A**) and functional (**B**) annotations of 20 coral metagenomes. Genus level taxonomic annotation was performed using FOCUS. Functional annotation of level 3 subsystems was performed using SUPER-FOCUS using blastx and DB_98

For the taxonomic profiling, Fig. 12a was generated using SciPy (Jones *et al.*, 2001) and matplotlib (Hunter, 2007) (both open source python programming language libraries), and it shows the hierarchical clustering based on the pairwise Euclidean distances of the relative genus abundance of the each coral metagenome. The clustering shows that the samples from the same site cluster together, which suggest they have a similar microbial community.

For the functional profiling, the same hierarchical clustering approach, using the distances between the relative abundances of the level 1, 2 and 3 subsystem classification were used. Figure 12b also shows that samples from the same sites had similar subsystem level 3 profiles (Supplementary Fig. S9 for levels 1 and 2). Both figures show that there is a microbial core that is preserved on some of the reefs; however, the nature of that core differs between taxonomy and function, as we have shown before (Dinsdale *et al.*, 2008). This trend suggests that the organisms are quite stable at different sites while the functions change to allow adaptation to local environments.

### 3.5 Final considerations

FOCUS is integrated with a SUPER-FOCUS pipeline which permits the tool to also report the taxonomic profile for a given metagenomic dataset. Both functional and taxonomic profiles are also provided by MEGAN and MG-RAST; RTMg only reports the functional assignments. SUPER-FOCUS provides three advances in the functional profiling compared with other tools: (i) It uses a fast aligner; (ii) it uses clustered databases in order to obtain a fast profile with little loss of sensitivity and (iii) focuses on the microbes present in the input data to attain a more microbial profile. As a default the tool, uses three advances; however, the user can select any combination of options.

## 4 Conclusions

Here, we present SUPER-FOCUS, an agile solution to identify the subsystems present in metagenomic samples that first determines the taxonomic composition of the entire metagenome by using FOCUS, and uses this knowledge to create, on the fly, a reduced database only containing the subsystems present in the organisms found. This makes SUPER-FOCUS a faster and still accurate tool to profile the functional subsystems in metagenomes. SUPER-FOCUS reports very similar results to currently available tools, but does so faster and using less memory.

## Supplementary Material

Supplementary Data
