# Supplementary material for: SUPER-FOCUS: a tool for agile functional analysis of shotgun metagenomic data
Source: Bioinformatics. 2015 Oct 9;32(3):354–61. doi: 10.1093/bioinformatics/btv584 (PMC4734042; doi:10.1093/bioinformatics/btv584)
Supplement: Supplementary Data [file supp_32_3_354__index.html]

SUPER-FOCUS: a tool for agile functional analysis of shotgun metagenomic data — Supplementary Data 

# SUPER-FOCUS: a tool for agile functional analysis of shotgun metagenomic data

## Supplementary Data

files

- Supplementary Data - zip file
