# Supplementary figures and images for: SUPER-FOCUS: a tool for agile functional analysis of shotgun metagenomic data
Source: Bioinformatics. 2015 Oct 9;32(3):354–61. doi: 10.1093/bioinformatics/btv584 (PMC4734042; doi:10.1093/bioinformatics/btv584)

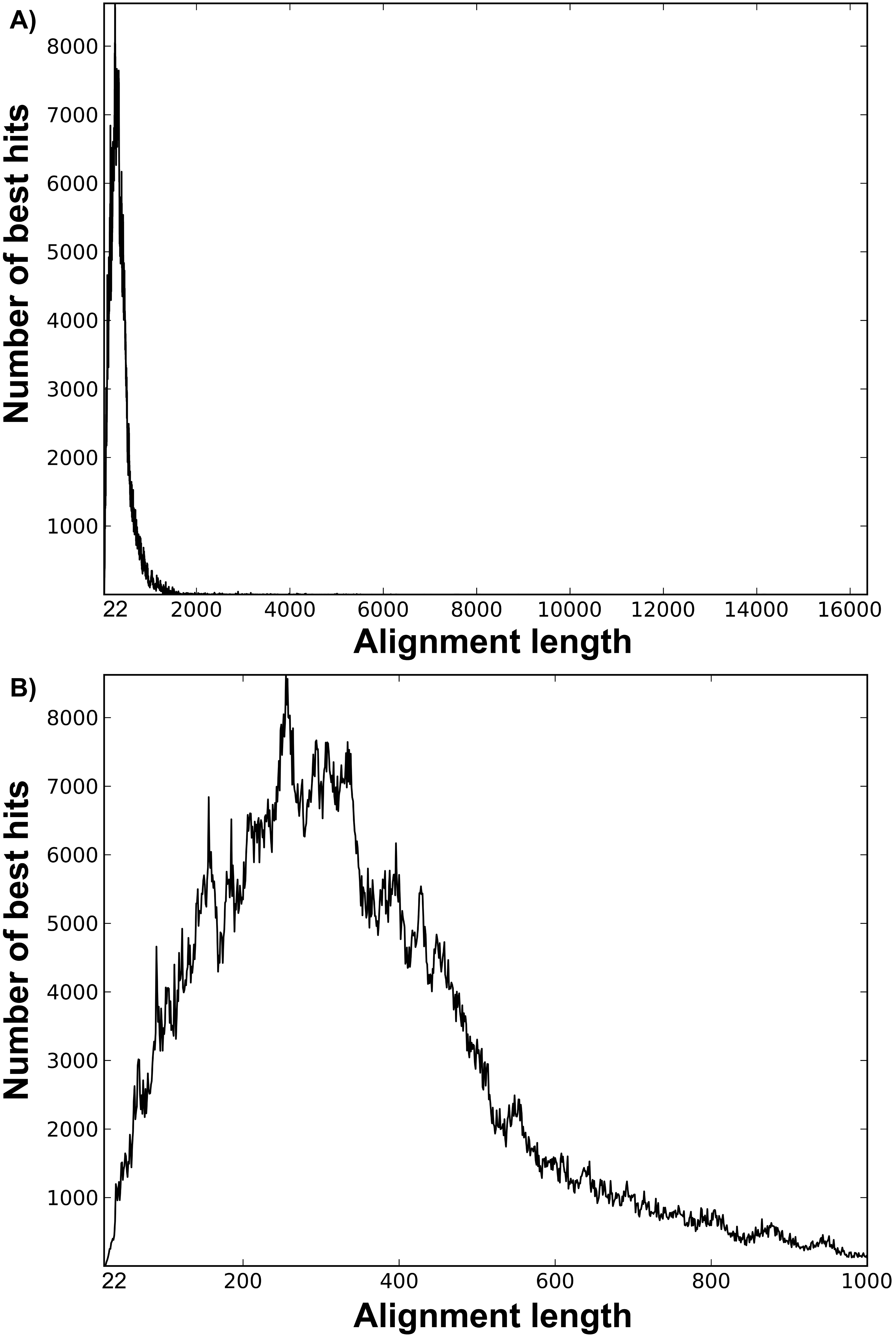

Supplement: Supplementary Data [file supp_btv584_suppl_data.zip › Supplementary_Figure_1.jpg]

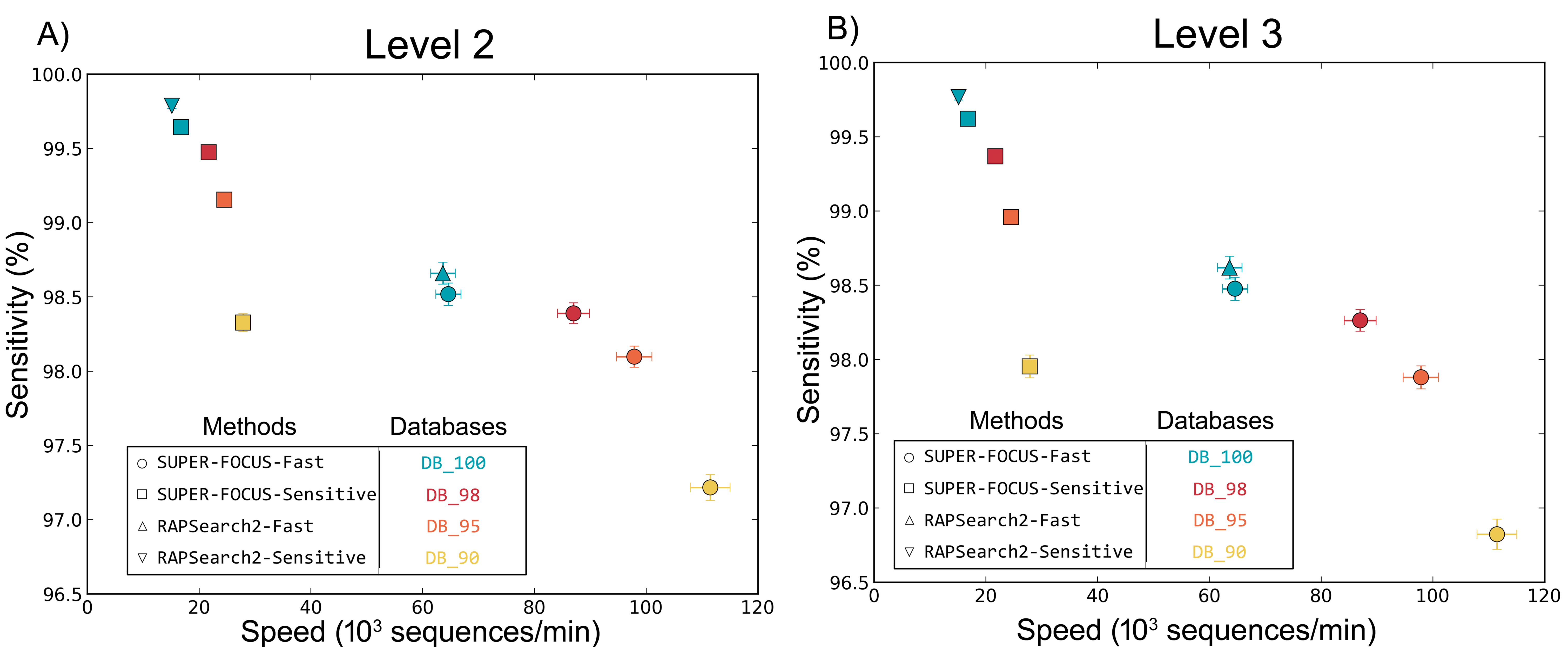

Supplement: Supplementary Data [file supp_btv584_suppl_data.zip › Supplementary_Figure_2.png]

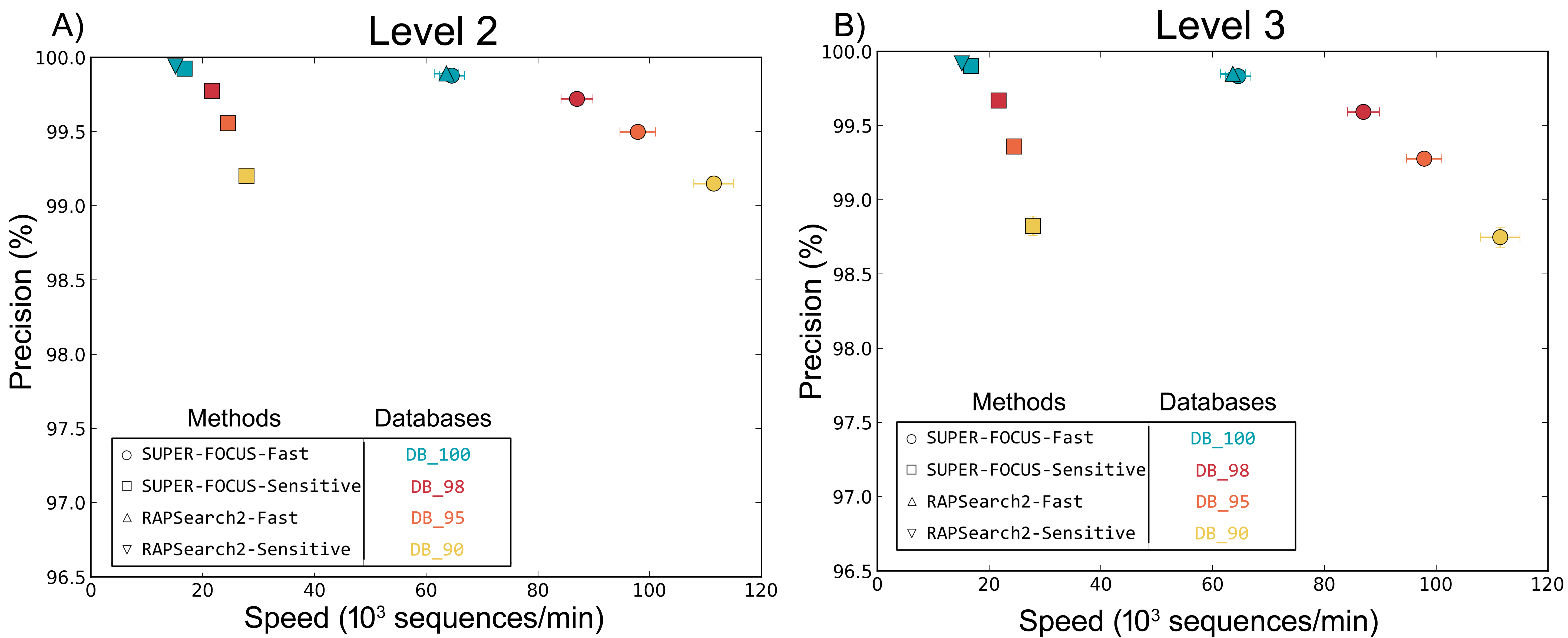

Supplement: Supplementary Data [file supp_btv584_suppl_data.zip › Supplementary_Figure_3.png]

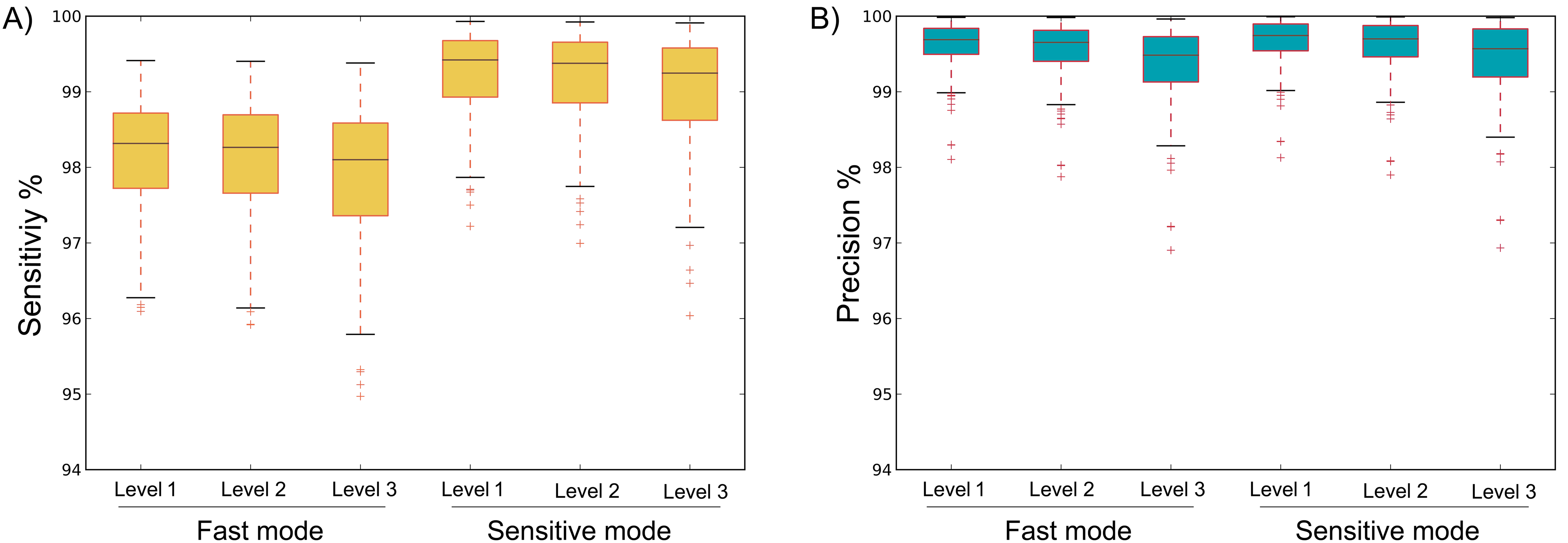

Supplement: Supplementary Data [file supp_btv584_suppl_data.zip › Supplementary_Figure_4.png]

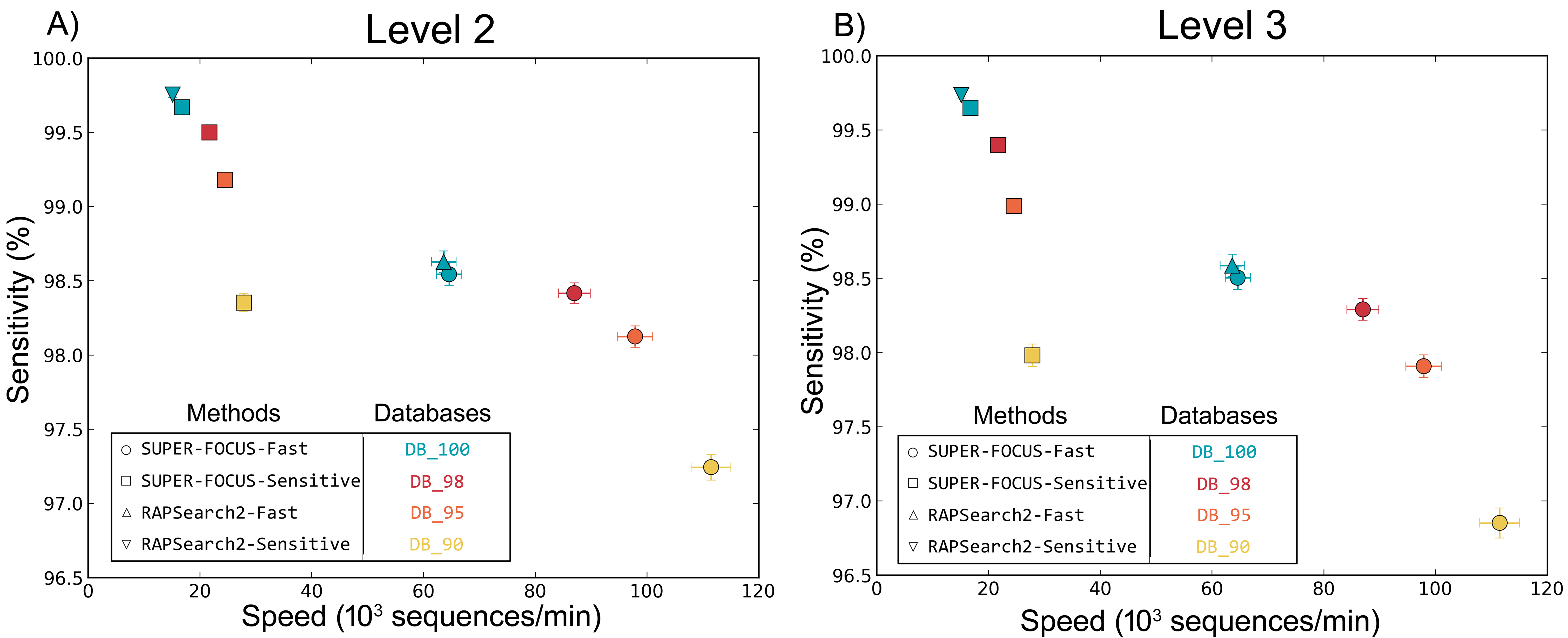

Supplement: Supplementary Data [file supp_btv584_suppl_data.zip › Supplementary_Figure_5.png]

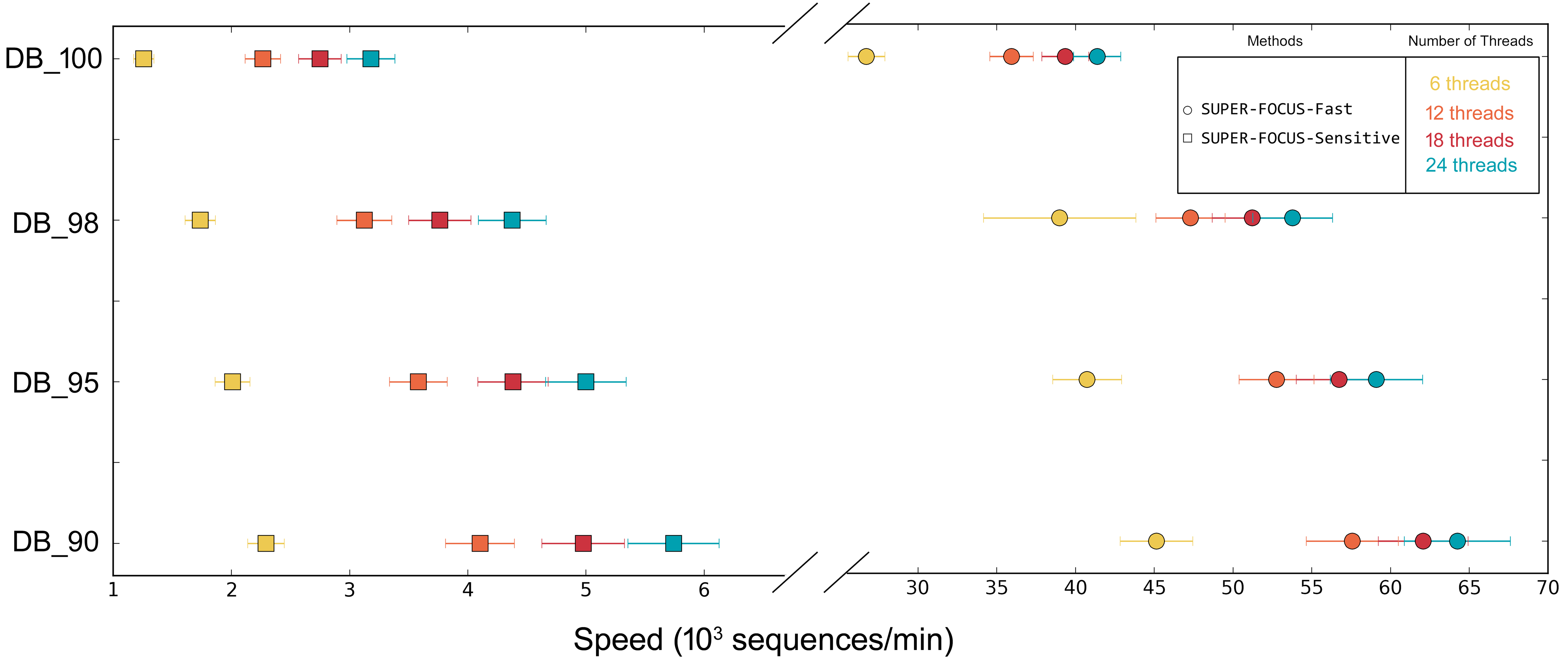

Supplement: Supplementary Data [file supp_btv584_suppl_data.zip › Supplementary_Figure_6.png]

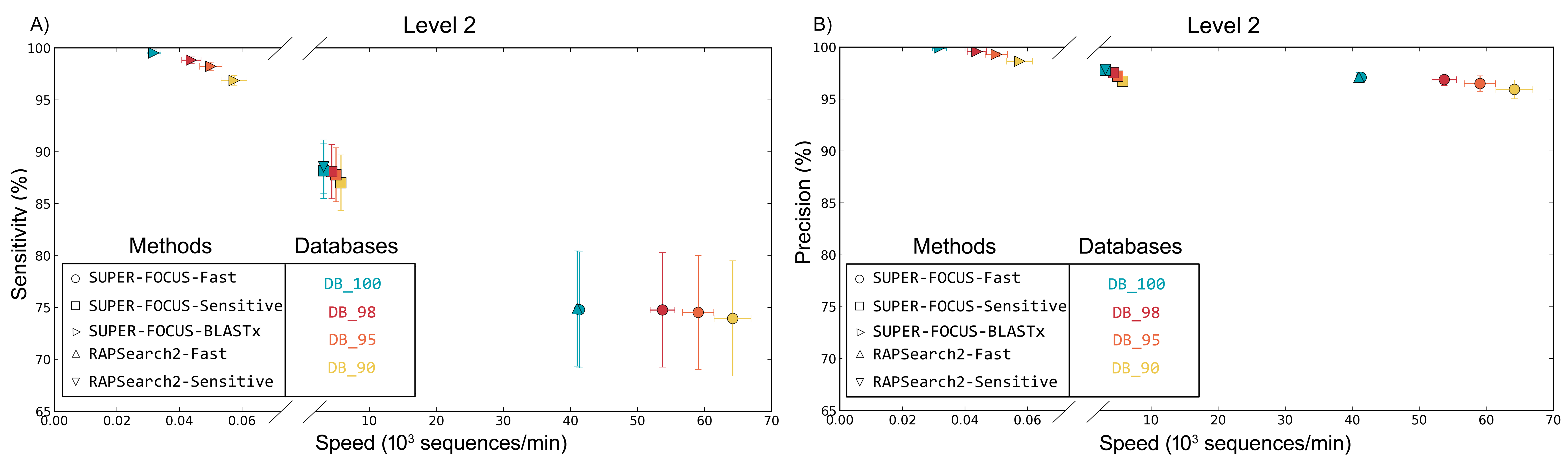

Supplement: Supplementary Data [file supp_btv584_suppl_data.zip › Supplementary_Figure_7.png]

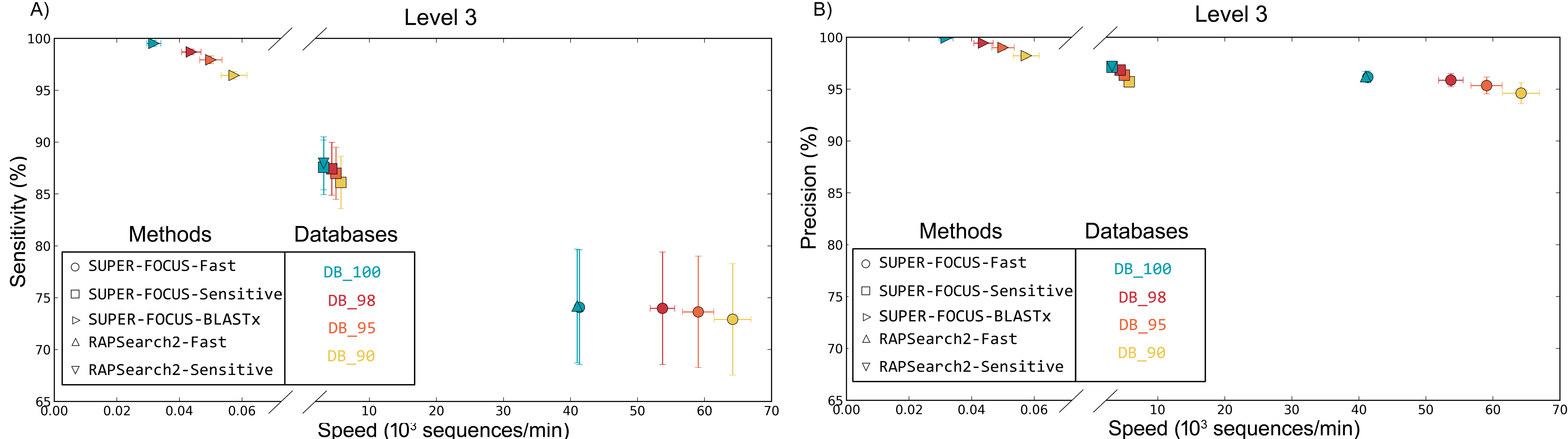

Supplement: Supplementary Data [file supp_btv584_suppl_data.zip › Supplementary_Figure_8.png]

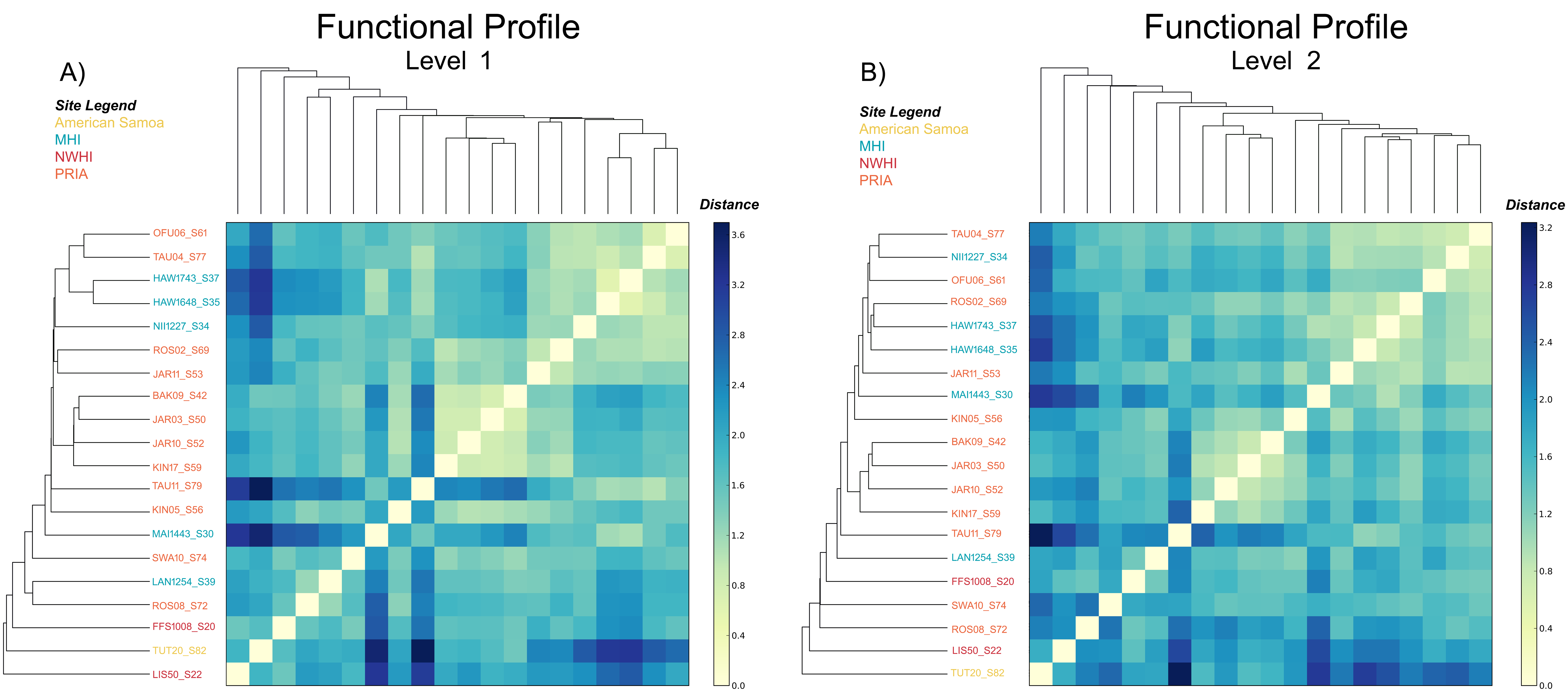

Supplement: Supplementary Data [file supp_btv584_suppl_data.zip › Supplementary_Figure_9.png]
