# Supplementary material for: SUPER-FOCUS: a tool for agile functional analysis of shotgun metagenomic data
Source: Bioinformatics. 2015 Oct 9;32(3):354–61. doi: 10.1093/bioinformatics/btv584 (PMC4734042; doi:10.1093/bioinformatics/btv584)
Supplement: Supplementary Data [file supp_btv584_suppl_data.zip › Supplementary_Material.doc]

# 1 Suplementary methods

## 1.1 The presence or absence of subsystems in complete genomes using RAST

We downloaded all the complete genomes for the organisms used in section 2.3 and annotated them using RAST (Azi*z et a*l., 2008). Genomes were uploaded into RAST via the SEED API (Dis*z et a*l., 2010) using the 'submit_RAST_job' perl script and subsystems present in each genome were retrieved via the 'retrieve_RAST_job' script.	The RAST presence/absence table for subsystems present into each genus was created as described in the second paragraph of section 2.3.

# 2 Suplementary results AND DISCUSSION

## 2.1 Comparison between the RAST and blastp presence/absence

We compared the two tables by looking into the % of agreement among the assignments for presence or absence of a subsystem into all genera. It was found that the two tables agreed in ~78.7% of the assignments, 11.4% of assignments were only present in the RAST table, and 9.89% of assignments in the blastp table.

In addition, 50 metagenomes used in the manuscript from the Human Microbiome Project (HMP) (Consortium, 2012) were profiled using FOCUS (Silv*a et a*l., 2014), and the subsystems present in these organisms were predicted using the RAST and blastp presence/absence tables.

The predicted subsystems to be into the 50 metagenomes were compared to the subsystems found by the blastx assignments (considered in section 3.1 of the manuscript to be the "right answer") using the complete SEED (DB_100), and the results shows that ~98% ± 1.36 of the subsystems predicted using the blastp table agreed with the blastx assignments and 94.15%±1.89 for agreement for the RAST table, which shows that there is no major difference between the two tables, and they would produce a similar result for the functional profiling.

**References**

Aziz,R.K. *et al.* (2008) The RAST Server: rapid annotations using subsystems technology. *BMC Genomics*, **9**, 75.

Consortium,T.H.M.P. (2012) Structure, function and diversity of the healthy human microbiome. *Nature*, **486**, 207–214.

Disz,T. *et al.* (2010) Accessing the SEED genome databases via Web services API: tools for programmers. *BMC Bioinformatics*, **11**, 319.

Silva,G.G.Z. *et al.* (2014) FOCUS: an alignment-free model to identify organisms in metagenomes using non-negative least squares. *PeerJ*, **2**, e425.
